# Environmental Contamination with *Candida* Species in Multiple Hospitals Including a Tertiary Care Hospital with a *Candida auris* Outbreak

**DOI:** 10.20411/pai.v4i2.291

**Published:** 2019-10-28

**Authors:** Jessica Kumar, Brandon Eilertson, Jennifer L. Cadnum, Chauna S. Whitlow, Annette L. Jencson, Nasia Safdar, Sarah L. Krein, Windy D. Tanner, JeanMarie Mayer, Matthew H. Samore, Curtis J. Donskey

**Affiliations:** 1 Geriatric Research, Education, and Clinical Center; Louis Stokes Cleveland VA Medical; Cleveland, Ohio; 2 SUNY Downstate Medical Center; Brooklyn, New York; 3 Research Service; Louis Stokes Cleveland VA Medical Center; Cleveland, Ohio; 4 Pathology and Laboratory Medicine Service; Louis Stokes Cleveland VA Medical Center; Cleveland, Ohio; 5 Infectious Disease Division; University of Wisconsin-Madison School of Medicine and Public Health; Madison, Wisconsin; 6 William S. Middleton Memorial Veterans Hospital; Madison, Wisconsin; 7 VA Ann Arbor Healthcare System; Ann Arbor, Michigan; 8 University of Utah School of Medicine; Division of Epidemiology; Salt Lake City, Utah; 9 Case Western Reserve University School of Medicine; Cleveland, Ohio

**Keywords:** Candida auris, Candida species, environmental contamination, infection control, floor and sink drain disinfection

## Abstract

**Background:**

Environmental sources have been implicated as a potential source for exogenous acquisition of *Candida* species, particularly the emerging multidrug-resistant *Candida auris*. However, limited information is available on environmental reservoirs of *Candida* species in healthcare facilities.

**Methods:**

During a 6-month period, cultures for *Candida* species were collected from high-touch surfaces in patient rooms and from portable equipment in 6 US acute care hospitals in 4 states. Additional cultures were collected from sink drains and floors in one of the hospitals and from high-touch surfaces, portable equipment, and sink drains in a hospital experiencing an outbreak due to *C. auris. Candida* species were identified using matrix-assisted laser desorption/ionization time-of-flight mass spectometry.

**Results:**

*Candida* species were recovered from patient rooms in 4 of the 6 hospitals. Seven of 147 patient room cultures (4.8%) and 1 of 57 (1.8%) portable equipment cultures were positive, with the most common species being *C. parapsilosis.* For the hospital where additional sites were sampled, *Candida* species were recovered from 8 of 22 (36.4%) hospital room floors and 4 of 17 (23.5%) sink drains. In the facility with a *C. auris* outbreak, *Candida* species were frequently recovered from sink drains (20.7%) and high-touch surfaces (15.4%), but recovery of *C. auris* was uncommon (3.8% of high-touch surfaces, 3.4% of sink drains, and 0% of portable equipment) and only present in rooms that currently or recently housed a patient with *C. auris*.

**Conclusion:**

*Candida* species often contaminate surfaces in hospitals and may be particularly common on floors and in sink drains. However, *C. auris* contamination was uncommon in a facility experiencing an outbreak, suggesting that current cleaning and disinfection practices can be effective in minimizing environmental contamination.

## INTRODUCTION

*Candida* species are an important cause of invasive infections in healthcare settings, particularly in immunocompromised and critically ill patients [1, 2]. Although most infections due to *Candida* species are endogenous, exogenous acquisition may also occur in healthcare facilities [1, 3-14]. In a recent study of the molecular epidemiology of candidemia in Iceland, molecular typing with polymerase chain reaction fingerprinting suggested that 19% to 40% of all infections were part of nosocomial clusters not identified by routine hospital surveillance [12]. The hands of healthcare personnel are considered the most important source of patient-to-patient transmission of *Candida* species [1, 4, 7]. Hand contamination with healthcare-associated pathogens may be acquired through direct contact with colonized or infected patients or after contact with contaminated surfaces [15, 16]. Some studies have implicated environmental sources, including contaminated portable equipment, as a source for acquisition of *Candida* species. Sanchez *et al* [4] demonstrated that a *Candida parapsilosis* strain causing infections was recovered from inanimate surfaces in a new intensive care unit before patients were admitted. The emerging fungal pathogen *Candida auris* has frequently been recovered from environmental surfaces in rooms of colonized or infected patients [13], and a recent report of an outbreak of *C. auris* in an intensive care unit linked transmission to shared temperature probes [14]. The ability of *C. auris, C. parapsilosis*, and *Candida glabrata* strains to survive for prolonged periods on dry and moist surfaces may increase the likelihood of transmission from the environment [17].

Given the importance of *Candida* species as healthcare-associated pathogens, there is a need for additional studies of potential environmental reservoirs. In a single hospital, we previously reported that *Candida* species were recovered frequently from the environment, particularly from moist surfaces [17]. Moreover, we found that *Candida* species exhibited relative resistance to widely used quaternary ammonium disinfectants and ultraviolet light [18, 19]. Here, we conducted a culture survey in 6 hospitals to assess the frequency of recovery of *Candida* species from high-touch surfaces in patient rooms. To obtain information on contamination of sites such as floors and sink drains, we conducted additional culture surveys in 2 hospitals, including a facility experiencing a *C. auris* outbreak.

## METHODS

### Protection of Human Research Participants

The institutional review boards of the 6 facilities participating in the multihospital survey approved the study protocol. For the facility experiencing the *Candida auris* outbreak, collection of environmental cultures was conducted as a quality improvement project to assess the efficacy of cleaning and disinfection practices. No information that could identify patients was collected.

### Multihospital Assessment of Environmental Contamination With *Candida* Species

During a 6-month period from April 2016 to June 2017, environmental cultures for multiple healthcare-associated pathogens were collected in 6 US acute care hospitals in 4 states. The facilities used sporicidal disinfectants for high-touch surfaces in rooms of patients with *Clostridioides difficile* infection (CDI), non-sporicidal disinfectants for other patient rooms, and detergents for floors. The facilities included 2 tertiary care medical centers and 4 Veterans Affairs Medical Centers. A detailed description of the study and the results of bacterial cultures will be presented elsewhere. Samples from occupied contact precautions rooms and non-isolation rooms were cultured on multiple general medical/surgical wards or intensive care units, with culture collections occurring at each facility every 2 weeks for 8 to 11 cycles. Up to 4 contact precautions rooms (depending on availability) and an equal number of non-isolation rooms were sampled during each culture collection.

For each patient room, composite cultures were collected from standardized sites ≤3 feet from the patient (bed rail, bedside table, telephone, and call button), >3 feet from the patient inside the room (room inner door handle, charting bar code scanner and keyboard, and the bed control panel), and in the bathroom (toilet grab bar, toilet flush handle, toilet rinse spout handle, and inner bathroom door handle). If the room occupant was using a commode, the grab handles and commode seat were sampled in place of the bathroom samples. Standardized surfaces from shared equipment were also sampled in each unit. The shared unit equipment sample was a composite of surfaces from a glucometer, bladder scanner probe handle, and Doppler ultrasound or portable vital signs monitor.

All cultures were collected using cellulose sponges (Sponge Stick with neutralizing buffer, 3M). The entire surface area of each culture site was contacted with the Sponge Stick which was pre-moistened with neutralizing buffer. The specimens were processed as previously described [20-22]. After completion of bacterial cultures, the specimens were frozen at −80°C in 15% glycerol as a cryoprotectant and thawed for *Candida* species cultures; 500 µL of each sample was plated onto Sabouraud dextrose agar and incubated for 96 hours at 37°C. Colonies consistent with *Candida* species were subjected to identification using matrix-assisted laser desorption/ionization time-of-flight (MALDI-TOF) mass spectrometry (Bruker Biotyper CA System, Bellerica, MA). Preliminary studies indicated that freezing of the specimens did not significantly reduce the yield of *Candida* species (data not shown).

### Additional Environmental Cultures in One of the Study Hospitals to Assess Sink Drain and Floor Contamination

A recent study demonstrated that sink drains may be a potential source for dispersal of *Candida* species [22]. It has also been suggested that hospital floors might be an underappreciated source for dissemination of healthcare-associated pathogens [23, 24]. Therefore, we collected an additional set of cultures in one of the study hospitals to obtain information on contamination of sink drains and floors. The facility was a Veterans Affairs Medical Center. The facility used a sporicidal disinfectant for high-touch surfaces in rooms of patients with CDI, a non-sporicidal disinfectant for other patient rooms, and a detergent for floors. Sink bowls were routinely disinfected during post-discharge room cleaning and disinfection, but no sink drain disinfection was performed. The cultures were collected using BBL Culture Swabs (Becton Dickinson, Cockeysville, MD). For floors, pre-moistened swabs were used to sample a total area of 20×20 cm from sites adjacent to the bed, adjacent to the trash can, and in the bathroom. For sink drains, the swabs were inserted through the strainer and samples were collected from the surface of the drain pipes to a depth of 1 inch below the strainer. For comparison, a composite of the high-touch surfaces <3 feet from the patient (described previously) was sampled with a single pre-moistened swab. The swabs were plated directly onto Sabouraud dextrose agar. The cultures were processed as described previously and colonies consistent with *Candida* species were identified using MALDI-TOF.

### *Candida* Species Environmental Contamination in a Hospital Experiencing a *C. auris* Outbreak

We collected environmental cultures in a 376-bed tertiary care hospital located in Brooklyn, New York which was experiencing a *C. auris* outbreak when the cultures were collected. The hospital experiencing the *C. auris* outbreak provided care for a total of 8 patients with *C. auris*, including 3 with positive clinical cultures (1 with candidemia and 2 with positive urine cultures that were deemed asymptomatic funguria) and 5 with colonization identified by culturing samples from the groin and axilla. The first patient was admitted in September, 2017, but carriage of *C. auris* was not recognized until November, 2017. A second patient was admitted in January, 2018 and developed candidemia in February, 2018. In May-June, 2018, 6 colonized patients were detected through screening. The final identified patient with *C. auris* was discharged during the last week of June, 2018.

An initial set of environmental cultures was obtained in May, 2018. During this period, routine cleaning and disinfection of patient rooms included use of a quaternary ammonium disinfectant once daily for disinfection of high-touch surfaces and a bleach wipe product for post-discharge cleaning and disinfection. In *C. auris* rooms, bleach wipes were used for once-daily disinfection of high-touch surfaces and floors were cleaned with a detergent; sink bowls were disinfected during post-discharge room cleaning and disinfection, but no sink drain disinfection was performed. At the time the initial cultures were collected, 3 patients colonized with *C. auris* were in the facility, including 2 in a 2-bed room. The rooms of the *C. auris* patients and other rooms that had previously housed *C. auris* patients were included in the culture survey. A second set of cultures was obtained in June, 2018 after switching to a chlorine-based spray disinfectant for routine cleaning and disinfection of all patient rooms; the frequency of daily disinfection in *C. auris* rooms was also increased to 3-times daily. Three patients colonized with *C. auris* were in the facility at the time of the second set of cultures, and their rooms were included in the culture survey.

The cultures were collected using BBL Culture Swabs as described previously. For sink drains, the swabs were inserted through the strainer and samples were collected from the surface of the drain pipes to a depth of 1 inch below the strainer. For high-touch surfaces and portable equipment, 5×10 cm areas of larger surfaces were sampled and the entire surface area of smaller items (eg, call buttons, telephones) was sampled. The swabs were plated directly onto Sabouraud dextrose agar. The cultures were processed as described previously and colonies consistent with *Candida* species were identified using MALDI-TOF.

## RESULTS

### Multihospital Assessment of Environmental Contamination With *Candida* Species

**Figure 1.**
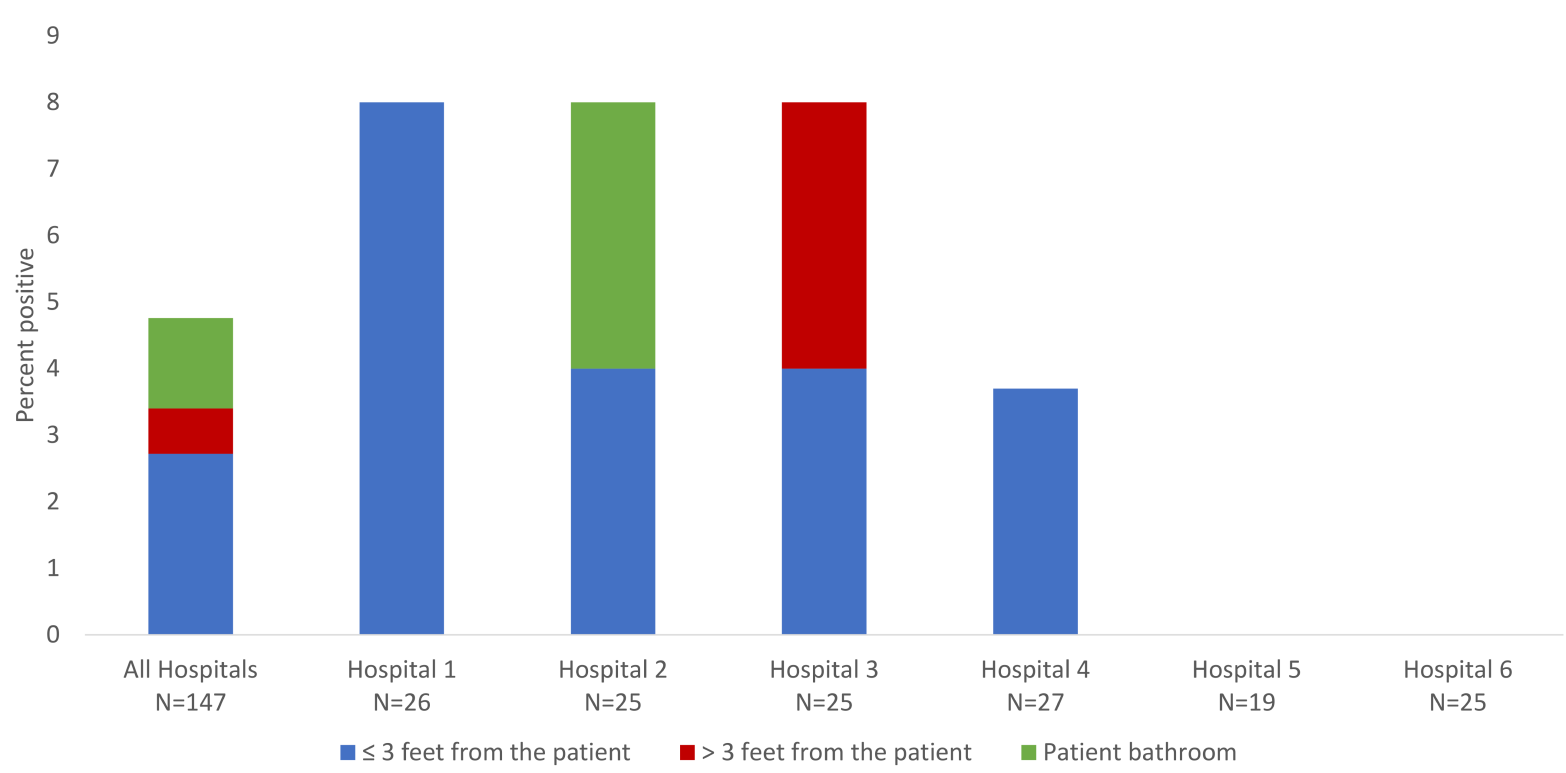
Frequency of recovery of *Candida* species from environmental surfaces in patient rooms in 6 hospitals.

Figure 1 shows the percentage of patient rooms with positive cultures for *Candida* species from high-touch surfaces in the 6 study hospitals and the sites of recovery. *Candida* species were recovered from 1 or more rooms in 4 of the 6 hospitals. Overall, *Candida* species were recovered from 7 of 147 rooms (4.8%); 8 total sites were positive (ie, 1 contaminated room was positive both ≤3 feet and >3 feet from the patient). The *Candida* species recovered included *Candida parapsilosis* (N=5), *Candida lusitaniae* (N=3), and *Candida guilliermondii* (N=1). Contamination was detected in 4 (3.1%) rooms ≤3 feet from the patient, 1 (0.7%) room >3 feet from the patient, and 2 (1.4%) bathrooms. The frequency of recovery of *Candida* species was similar for contact precautions rooms and non-isolation rooms (3 of 67, 4.5% versus 4 of 80, 5.0%). *Candida* species were recovered from 1 of 57 (1.8%) sets of cultures of portable equipment cultured in the 6 hospitals.

### Additional Cultures in One of the Study Hospitals to Assess Sink Drain and Floor Contamination

Additional cultures were collected from the hospital labeled hospital 1 in Figure 1. *Candida* species were recovered from 8 of 22 (36.4%) hospital room floors, 4 of 17 (23.5%) sink drains, and 2 of 22 (9.1%) high-touch surfaces. The species recovered from floors included *C. parapsilosis, C. metapsilosis, C. orthopsilosis, C. glabrata*, and *C. albicans*. The species recovered from sink drains included *C. parapsilosis* and *C. tropicalis*. The species recovered from high-touch surfaces included *C. parapsilosis, C. lusitaniae*, and *C. guillermondii*. The number of colony-forming units of *Candida* species recovered from sink drains (mean=90, range 8 to 300 CFU) was higher than the number recovered from floors or high-touch surfaces (mean=2, range 1 to 4 CFU).

### *Candida* Species Contamination in a Hospital Experiencing a *C. auris* Outbreak

Table 1 shows the results of cultures collected from the hospital experiencing the *C. auris* outbreak. The percentages of cultures positive for *Candida* species were similar for cultures collected during the disinfectant protocols 1 and 2. However, *C. auris* was only recovered from high-touch surfaces during disinfectant protocol 1 when a quaternary ammonium disinfectant was used once-daily in non-*C. auris* rooms and bleach wipes were used once-daily and post-discharge in *C. auris* rooms. One sink drain was positive for *C. auris* during disinfectant protocol 2 when a chlorine-based disinfectant was used for routine cleaning and disinfection of all patient rooms and 3-times daily and post-discharge in *C. auris* rooms. No portable equipment cultures were positive for *C. auris*. The high-touch surfaces with positive cultures for *C. auris* included a bedrail/bedside table and a call button/telephone.

**Table 1.** Recovery of *Candida* species from environmental cultures in an acute care hospital experiencing an outbreak of *Candida auris* during periods when different disinfectant protocols were used

	Disinfectant Protocol 1* (May, 2018)		Disinfectant Protocol 2** (June-July, 2018)	
Culture site	No. positive/No. collected (%)	*Candida* species recovered	No. positive/No. collected (%)	*Candida* species recovered
Sink drains	3/17 (17.6)	*C. tropicalis* (N=2) *C. albicans* (N=1)	3/12 (25.0)	*C. auris* (N=1) *C. lusitaniae* (N=2) *C. intermedia* (N=1)
High-touch surfaces	4/30 (13.3)	*C. auris* (N=2) *C. parapsilosis* (N=1) *C. tropicalis* (N=1)	4/22 (18.2)	*C. albicans* (N=2) *C. parapsilosis* (N=1) *C. lusitaniae* (N=1)
Portable equipment	0/13 (0)	None	1/32 (3.1)	*C. parapsilosis* (N=1)

*, Disinfectant Protocol 1: routine cleaning and disinfection of patient rooms included use of a quaternary ammonium disinfectant once daily for disinfection of high-touch surfaces and a bleach wipe product for post-discharge cleaning and disinfection; in *C. auris* rooms bleach wipes were used for once-daily disinfection of high-touch surfaces.

**, Disinfectant Protocol 2: routine cleaning and disinfection of patient rooms included use of a chlorine-based spray disinfectant for routine cleaning and disinfection of all patient rooms; the frequency of daily disinfection in *C. auris* rooms was increased to 3-times daily.

## DISCUSSION

Although many studies have investigated contamination of the healthcare environment with bacterial pathogens, little information is available on the frequency of contamination with *Candida* species. In 6 hospitals, we recovered *Candida* species from high-touch surfaces in 4.8% of patient rooms and from 1.8% of shared portable equipment. In one of the hospitals, additional cultures demonstrated frequent recovery of *Candida* species from sink drains (23.5%) and floors in patient rooms (36.4%). It is likely that more frequent contamination may have been detected on high-touch surfaces if rooms of patients with known *Candida* species colonization or infection had been sampled.

In a hospital experiencing an outbreak of *C. auris, Candida* species were frequently recovered from sink drains (20.7%) and high-touch surfaces (15.4%). However, *C. auris* was rarely recovered (3.8% of high-touch surfaces, 3.4% of sink drains, and 0% of portable equipment) and only from sites in rooms that currently or recently housed a patient colonized or infected with *C. auris*. It is notable that *C. auris* was recovered from high-touch surfaces during the period when once-daily bleach wipe disinfection was performed in *C. auris* rooms, but not when the frequency of disinfection was increased to 3-times daily with a chlorine-based spray disinfectant. Although the conclusions that can be drawn from these findings are limited, the data suggest that current cleaning practices including frequent application of a sporicidal disinfectant in *C. auris* rooms can be effective in minimizing environmental contamination. The finding of a positive sink drain culture during the second point-prevalence culture survey does raise some concern of the potential for sink drains to be a reservoir for *C. auris* persistence which is not amenable to cleaning and disinfection. It has been demonstrated that *Candida* species colonizing sink drains may disperse to countertops by splashing of water during sink operation [22].

We found that *Candida* species contamination was more common on high-touch surfaces in close proximity to patients (3.1% contamination on surfaces <3 feet from the patient and 0.7% on surfaces >3 feet from the patient). Surfaces in close proximity to patients may be more frequently contaminated than more distant surfaces in patient rooms [25, 26]. In addition, it has been demonstrated that during procedures shedding of methicillin-resistant *Staphylococcus aureus* (MRSA) by colonized patients occurs significantly more often on surfaces less than 3 feet versus greater than 3 feet from the patient [27]. Therefore, it has been proposed that personnel remaining in more distant lower-risk areas of contact precautions rooms might not be required to wear personal protective equipment if they do not contact surfaces [25, 26].

One striking finding from our study was the high frequency of floor contamination with *Candida* species in the single hospital where floors were sampled. This finding is consistent with previous studies that have demonstrated that the floor is typically the most heavily contaminated environmental site in healthcare facilities [28]. The significance of floor contamination is uncertain. However, it has been suggested that hospital floors might be an underestimated source for dissemination of healthcare-associated pathogens because organisms on floors can be transferred from shoes or socks or other high-touch items to the hands of patients or personnel [23, 24, 28].

Our study has some limitations. We did not have information on whether the patients in the study rooms were colonized or infected with *Candida* species. Studies are needed to assess environmental contamination in rooms known to be occupied by colonized or infected patients, including on wards with patients at high risk for *Candida* species infection (eg, oncology wards). Our study does not provide information on whether environmental sites are contaminated through direct contact with colonized patients or via the hands of personnel. In addition, we did not confirm that *Candida* species in the environment can be transferred to patients or acquired on the hands of personnel. Previous studies have demonstrated that *C. difficile* and MRSA contamination on surfaces is frequently acquired on hands [15, 16]. Future studies are needed to determine if environmental isolates of *Candida* species are molecularly related to isolates that colonize or infect patients or are carried on the hands of personnel. Finally, floor cultures were collected in only a single hospital. Additional studies are needed to evaluate floor contamination with *Candida* species, particularly in facilities with *C. auris* outbreaks.

## CONCLUSION

Our findings demonstrate that *Candida* species often contaminate surfaces in hospitals and may be particularly common on floors and in sink drains. However, *C. auris* contamination was uncommon in a facility experiencing an outbreak, suggesting that current cleaning and disinfection practices can be effective in minimizing environmental contamination. Additional studies are needed to evaluate the role of contaminated high-touch surfaces in transmission of *Candida* species other than *C. auris*. Studies are also needed to investigate the potential for contaminated sink drains and floors to contribute to transmission.
